# Breed-Predispositions to Cancer in Pedigree Dogs

**DOI:** 10.1155/2013/941275

**Published:** 2013-01-17

**Authors:** Jane M. Dobson

**Affiliations:** Queen's Veterinary School Hospital, Department of Veterinary Medicine, University of Cambridge, Madingley Road, Cambridge CB3 OES, UK

## Abstract

Cancer is a common problem in dogs and although all breeds of dog and crossbred dogs may be affected, it is notable that some breeds of pedigree dogs appear to be at increased risk of certain types of cancer suggesting underlying genetic predisposition to cancer susceptibility. Although the aetiology of most cancers is likely to be multifactorial, the limited genetic diversity seen in purebred dogs facilitates genetic linkage or association studies on relatively small populations as compared to humans, and by using newly developed resources, genome-wide association studies in dog breeds are proving to be a powerful tool for unravelling complex disorders. This paper will review the literature on canine breed susceptibility to histiocytic sarcoma, osteosarcoma, haemangiosarcoma, mast cell tumours, lymphoma, melanoma, and mammary tumours including the recent advances in knowledge through molecular genetic, cytogenetic, and genome wide association studies.

## 1. Introduction

Cancer is an important disease in dogs and represents one of the major causes of canine death accounting for 27% of all deaths in purebred dogs in the UK in a recent mortality study [1]. This is slightly higher than what previously reported in a Danish Kennel club study (14.5% by Proschowesky et al.) and an earlier UK study (15.7% by Michell) but similar to a postmortem series of 2000 dogs, in which 23% of all dogs and 45% of dogs over 10 years of age died of cancer [2]. In the absence of reliable historical tumour registries, it is difficult to know whether the prevalence of cancer in dogs is actually increasing; however a number of factors may contribute to an increase in the diagnosis of cancer in dogs; as a result of improvements in health and welfare animals are living longer and cancer is generally a disease of older age [3]. Advances in veterinary medicine, particularly diagnostics and higher expectations of the pet owning public, are likely to result in an increased rate of diagnosis.

 As is the case in the human population, many different types of naturally occurring cancer may affect dogs and canine malignancies have been established as strong comparative models for the human disease due to their spontaneous development and frequency; dogs live in our environment and eat similar food and are thus exposed to similar risk factors, so the aetiology and pathogenesis of canine tumours is likely to be similar to that of human tumours [4–8]. A general comparison of the incidence of canine cancer with that of human cancer highlights some striking similarities and differences [9]. Breast cancer is the most common malignancy in women and the mammary gland is a common site for tumour development in bitches, although the risk is reduced in bitches spayed at a young age [10], demonstrating the importance of endogenous hormones in the development of this disease. In contrast, carcinomas of the prostate, a very common condition in men and also associated with hormonal stimulation, is relatively uncommon in dogs and occurs more frequently in neutered dogs [11]. Carcinomas of the lung and large bowel, the most common human tumours excluding breast and prostate, do not feature highly in the canine population, whereas soft tissue sarcomas, which are rare in humans, are relatively common in dogs.

**Figure 1:**
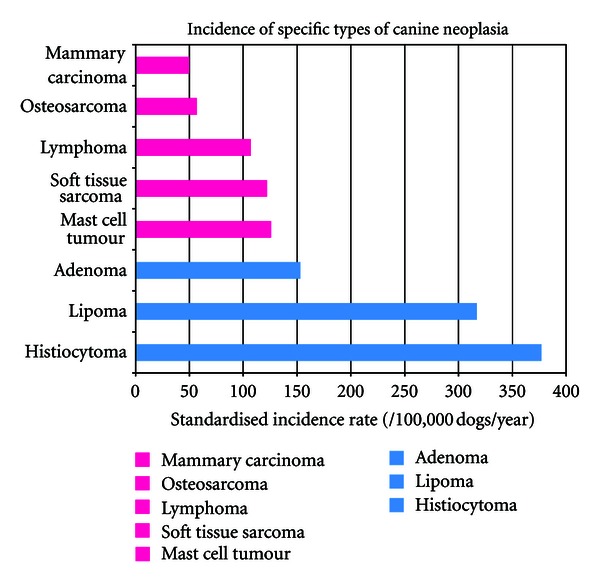
Incidence of specific types of canine neoplasia. From [12] (Pink bars denote malignant tumours, blue benign lesions).

However, whilst general trends for the occurrence of canine cancers are well established, accurate figures for the frequency of different types of tumours in the canine population are limited. One study of insured dogs from the United Kingdom showed the skin and soft tissues to be the most common sites for tumour-related claims (both benign and malignant) with a standardised incidence rate of 1437 per 100,000 dogs/year, followed by mammary, urogenital, lymphoid, endocrine, alimentary, and oropharyngeal sites. Canine cutaneous histiocytoma was the most common tumour type overall with a standardised incidence rate of 337 per 100,000 dog/year, followed by lipoma, adenoma, soft tissue sarcoma, mast cell tumour, and lymphoma (Figure 1, [12]). Other epidemiological studies, based on hospital populations or on surveys of intakes into pathology laboratories, are largely supportive of these estimates for the canine population as a whole [13–17], as are recent figures from a number of European Tumour Registries [18–20].

It is well recognised that differences exist between breeds of dog and their risk of developing certain types of cancer but there are few large scale epidemiological studies on the incidence of different types of cancer in the canine population which document the variation between breeds. The breeds with the highest proportional mortality for cancer in the Kennel Club/BSAVA study included the following, in descending order: Irish water spaniel, flat-coated retriever, Hungarian wirehaired vizsla, Bernese mountain dog, rottweiler, Italian spinone, leonberger, Staffordshire bull terrier, Welsh terrier, and giant Schnauzer ([1], Table 1). In a study of rates and causes of death in insured dogs in Sweden, Bonnett et al. [16] found that the Bernese mountain dog, Irish wolfhound, flat-coated retriever, boxer, and Saint Bernard were the five breeds of dog with the highest mortality from tumour-related death. The Bernese, Irish wolfhound, and leonberger were the top three in a subsequent examination of the same data base [21]. Bernese mountain dogs, flat-coated retrievers, golden retriever, and rottweilers were in the top 5 breeds with over 20% of deaths due to cancer in Denmark [22]. An owner- based questionnaire conducted in the UK segregated breeds into “overrepresented,” “average” and “underrepresented” with respect to dying of cancer and showed the same trends (Table 2) [23]. These population-based studies provide useful indicators of breeds at risk of cancer, but should not be regarded as completely definitive because the outcome often depends on the breed prevalence within the population at risk, which may explain the differences found in studies from different countries. The existence of other inherited diseases or breed-associated problems is a major confounding factor: for example the reason the bulldog has an apparent low risk of cancer in Table 1 may be due to its short life-expectancy due to other health issues that affect the breed [24]. Other limitations of such studies include owner compliance, bias through nonrandom sampling of the pedigree dog population, and accuracy of owner-reported cause of death. Furthermore a small number of dogs can seriously bias the results for numerically small breeds, as demonstrated by the red and white setter in Table 2 and probably the Hungarian wireheriad vizsla, and Welsh terrier in Table 1. However, the fact that all these studies consistently show similar overall breed-related predispositions to development of cancer has important implications in understanding the aetiology of cancer as it infers a genetic and heritable component.

The dog has a unique population structure with each breed arising from a limited number of founders. The dog is believed to have evolved from grey wolves possibly from Europe or the Middle East although interbreeding with local wolf populations clearly occurred elsewhere in the early history of dog lineages [25–27]. Although the wolf dog became domesticated 20–30,000 years ago, and dog types were gradually established for guarding, herding, and hunting purposes, it is only in the past 200 years or so that selective breeding practices have divided the dog population into over 300 discrete breeds worldwide. The English Kennel Club was established in the Victorian era to meet the demands of the dog breeding *Populus*, to regulate the registration of dog breeds, and to establish breed standards. Kennel clubs in Europe and America still impose strict standards on registration of pedigree dogs requiring that the ancestors of each dog must be registered as well. This combined with the frequent use of popular sires and inbreeding practices means that each breed is a closed, isolated population with virtually no gene flow between breeds [28]. Over the past 200 years this practice has resulted in reduced genetic diversity within breeds and greater genetic divergence between breeds. The average nucleotide heterozygosity when considered across dog breeds is comparable to the human population [29], but the level of genetic diversity within any single breed is considerably less than the species as a whole [25] indeed it has been estimated that whilst domestication of wild canid populations resulted in a 5% loss of nucleotide diversity, breed formation caused a 35% loss [30]. In many breeds the effective population size is very small even in normal times but in some breeds, for example, the Bernese mountain dog and the leonberger, genetic variation has been further reduced by serious population declines during war or hard economic times [31]. Mutations in a small number of genes of large effect are responsible for many breed characteristics; such selective breeding for exaggerated traits further reduces genetic diversity, and perhaps risks selection of mutations that predispose to disease [25, 32, 33]. Over 350 inherited disorders have been described in purebred dogs many of which are equivalent to human diseases [34, 35]. The limited genetic diversity seen in purebred dogs, facilitates genetic linkage or association studies on relatively small populations as compared to humans [36, 37] and by using newly developed resources, genome wide association studies in dog breeds are proving to be a powerful tool for unravelling complex disorders [38]. Although most forms of cancer are likely multifactorial in aetiology, the fact that different breeds of dog are predisposed to developing certain types of cancer (and conversely some breeds are at lesser risk) offers a unique opportunity to study and understand the genetic mechanisms underpinning cancer susceptibility [39, 40].

The purpose of this article is to review the current literature on predispositions to cancer in pedigree dogs, including the recent advances in knowledge through molecular genetic, cytogenetic, and genome wide association studies and to consider how the application of this new knowledge will inform our approach to the diagnosis, treatment, and possibly prevention of cancer in the future. 

**Figure 2:**
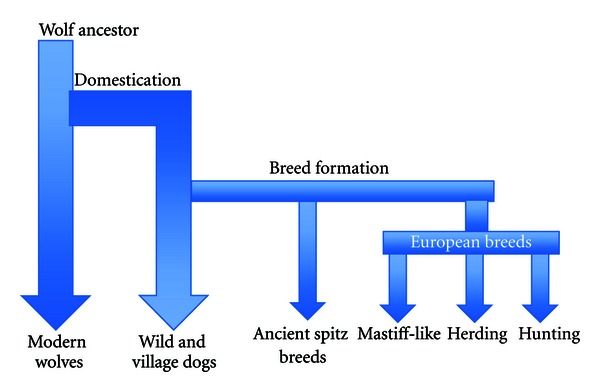
A simplified schematic summary of dog evolution: depicting the two evolutionary “bottle necks”: domestication and breed formation. It has been estimated that domestication of wild wolf-canine populations resulted in a 5% loss of nucleotide diversity, breed formation has caused a 35% loss [30].

**Figure 3:**
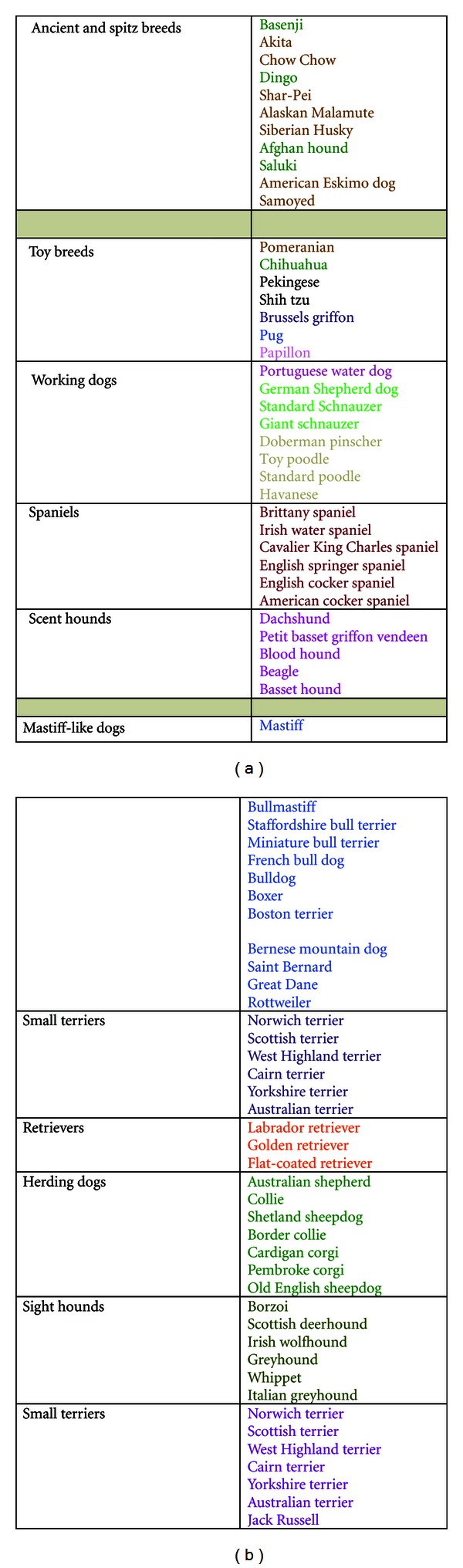
Summary of the neighbour-joining tree of domestic dogs [26] showing relationships between different breeds of dog on the basis of DNA analyses. Breeds that share either common behaviours or morphologic traits are grouped together on the basis of DNA analysis, indicating that they probably share common ancestors. The colours indicate breeds that probably share common founders.

Table 1, taken from the results of a health survey of purebred dogs in the UK [1], lists the proportional mortality for different breeds. The mean mortality rate to cancer for all breeds was 27%, thus those breeds recording greater than 30% deaths from cancer might be considered to be at greater risk or predisposed and those less than 25% cancer deaths, at reduced risk, although that is not to say that these latter breeds do not suffer particular types of cancer; for example, the Belgian shepherd has been documented to be at higher risk of gastric carcinoma [41] and the Scottish deerhound has been shown to have a heritable risk of osteosarcoma [42]. Considering the genetic structure of the purebred dog, phylogenetic analysis has shown separation of several breeds with ancient origin from a large group of breeds with presumed European origins. The former include the spitz type breeds: Shar-Pei, Shiba Inu, Chow Chow, Akita, Siberian Husky, and Alaskan Malamute. Further studies to characterise genetic variation within and among breeds established at least four distinct breed groupings: a subset of breeds with ancient Asian and African origins (as above but also including Basenji from Africa, Saluki, Afghan hound from the Middle East, and tibetan Terrier, Lhasa Apso from china), a group of Mastiff-like breeds, a group reflecting shared ancestral herding behaviour, and a group of hunting-type dogs (Figure 2) [43]. Most recently a neighbor-joining tree of domestic dogs showing the relationship among the various dog breeds has been constructed by genotyping 10 to 12 dogs for each of 80 breeds (Figure 3) [26]. Breeds that share either common behaviors or morphologic traits are grouped together on the basis of DNA analysis, indicating that they probably share common ancestors. In this context it is notable how some breed groupings “rank” in Table 1, with the Mastiff-type breeds dominating the top of the table and most of the ancient spitz type breeds below the mean. However, it is not possible to know whether this observation reflects genetic predisposition to cancer or body size or perhaps that the two are interrelated. Body size has previously been shown to correlate with longevity, with smaller breeds having a longer life expectancy [1, 23]. Of the breeds listed as over-represented in Tables 1 and 2, some have been associated with specific types of tumour, for example, Bernese mountain dog—systemic and malignant histiocytosis, Irish wolfhound—osteosarcoma, and others with a higher risk of more than one tumour type for example Boxer—mast cell tumour and brain tumour, Golden retriever—mast cell tumour, lymphoma, hemangiosarcoma. This observation also has important genetic implications, suggesting that some breeds may be like the rare human Li-Fraumeni families where a germ line mutation in a tumour suppressor gene (*TP53*) results in a hereditary predisposition to several types of cancer [44] or they may resemble the situation in families with mutations in *BRCA1* where the risk of developing breast and ovarian cancer is greater in relatives of affected people, rather than the cancer being transmitted as an autosomal dominant condition [45, 46]. In contrast other breeds affected by a specific type of tumour may have a more specific genetic abnormality leading to that particular type of tumour. For example, renal cystadenocarcinoma and nodular dermatofibrosis (RCND) is a rare inherited cancer syndrome in German shepherd dogs. Affected dogs develop bilateral multifocal tumours in the kidneys and multiple dense collagenous skin nodules [47]. In the dog the disease gene was mapped to a region on canine chromosome 5q12, corresponding to a gene encoding tumour-suppressor protein folliculin [48]. It was subsequently shown that the same gene is mutated in Birt-Hogg-Dube syndrome, a similar disease in humans [49].

For the purpose of this paper, breed associations will be considered by tumour type rather than by breed.

## 2. Histiocytic Sarcoma

Histiocytic sarcoma is the current term used to describe a spectrum of poorly differentiated, pleomorphic tumours shown to have an immunophenotype consistent with a myeloid dendritic antigen presenting cell origin: CD1+, CD4−, CD11c+, CD11d−, MHC II+, ICAM−1+, and Thy−1± [50]. As many of these reagents cannot be used on formalin fixed paraffin embedded sections, vimentin, MHC II, and the cell surface marker CD18 have become the standard panel for identification of histiocytic sarcoma [51, 52]. Localised histiocytic sarcoma describes those lesions which present as solitary masses, previously referred to as malignant fibrous histiocytoma [53–56], and disseminated histiocytic sarcoma, those that present with multifocal lesions, previously referred to as malignant histiocytosis [50]. Whilst histiocytic sarcoma is an uncommon tumour in the dog population as a whole, certain pedigree breeds appear predisposed to this disease, notably the Bernese mountain dog where the disseminated form of histiocytic sarcoma accounts for up to 25% of deaths in the breed [57] and the flat-coated retriever where the localised form is more common and accounts for approximately 25% of all tumours in the breed, and up to 50% of all malignancies [58, 59]. Disseminated histiocytic sarcoma in the Bernese typically presents with vague clinical signs including lethargy, anorexia, and weight loss. Investigations reveal multifocal disease primarily affecting the lungs, spleen, liver, bone marrow, and lymph nodes. Haematological abnormalities including anemia and thrombocytopenia are common [60–63]. The disease is rapidly progressive and fatal; many dogs are euthanized upon diagnosis; survival time from diagnosis has been reported to be 49 days [64]. In flat-coated retrievers localised lesions most commonly develop in the deep musculature of the limbs or in peri-articular sites; the elbow is the most common site [65]. Even these localised lesions are highly malignant with rapid dissemination to lymph nodes preceding haematogenous spread to parenchymal organs and the skin in over 70% of cases [66]. Thus both the localised and disseminated forms of histiocytic sarcoma are highly malignant conditions that are largely refractory to conventional treatments and carry a very poor prognosis. Treatment with Lomustine (CCNU) has been reported to result in some short term responses [56, 66–68]. The archetype of Bernese mountain dogs, being affected by the disseminated form of the disease, and flat-coated retrievers by the localised form, is not entirely accurate as up to 30% of histiocytic sarcomas in flat-coated retrievers are actually visceral, arising most commonly in the spleen with other visceral sites including liver, lung, mediastinum, and lymph nodes also affected [65]. Included in the visceral form affecting this breed is the hemophagocytic variant of histiocytic sarcoma, which arises from splenic or bone marrow derived macrophages and expresses CD11d [69, 70]. Furthermore localised periarticular histiocytic sarcoma is also recognised in the BMD, where it is possibly predisposed by joint disease [71]. Histiocytic sarcoma is not exclusive to the Bernese and flat-coated retriever; other breeds of dog where histiocytic sarcoma has been reported with some frequency include rottweilers and golden retrievers [50, 72–75].

The striking high incidence of histiocytic sarcoma in these breeds of dog suggests a heritable predisposition. A recent study of Danish Bernese mountain dogs described 13 dogs diagnosed with malignant histiocytosis, of which 11 were genealogically related [76]. In 1995 Padgett analysed the inheritance of “histiocytosis” in 127 affected Bernese mountain dogs and suggested a polygenic mode of inheritance and a calculated heritability of 0.298 [57]. More recently a pedigree of 327 Bernese mountain dogs (144 males, 183 females) was developed from 800 French and European dogs. A total of 121 dogs had a clinical diagnosis of histiocytic sarcoma. Detailed analysis of this pedigree showed that the segregation of the disease observed in these families could not be explained by a fully recessive model and that an oligogenic model was likely to be a better description of the genetic model underlying the disease [64]. Histiocytic sarcoma has recently been investigated by molecular cytogenetic profiling [77] and genome wide association studies [78]. Using genome wide array comparative genomic hybridization, supplemented with fluorescence *in situ* hybridization and loss of heterozygosity analysis, copy number aberrations (CNAs) were assessed in 146 histiocytic sarcomas, 101 from Bernese mountain dogs (68 from USA and 33 from France) and 45 from flat-coated retrievers (all from USA) [77]. Numerous CNAs were found, both gains and losses, throughout the genome. Thirty-one regions of the canine genome presented with recurrent CNAs of which 6 were highly recurrent, all of which were deletions located on dog chromosomes 2, 11, 16, 22, and 31. Almost all these recurrent CNAs were shared between the two breeds, suggesting that they are more associated with the cancer phenotype than with breed and a subset suggested involvement of known cancer-associated genes including deletions of the tumour suppressor genes *CDKN2A/B*, *RB1,* and *PTEN*. A small number of abberations were unique to each breed, and the authors speculated that these may contribute to the major differences in tumour location evident in the two breeds [77]. Interestingly dysregulation of *CDKN2* has also been associated with susceptibility to histiocytic sarcoma in Bernese mountain dogs by genome wide association study (GWAS) [78]. DNA was isolated from 474 blood samples from Bernese mountain dogs, 242 cases and 232 controls, 114 cases and 120 controls from North America and 128 cases and 112 controls from Europe. Both independent and combined GWAS were used to identify cancer-associated loci, fine mapping and sequencing narrowed the primary locus to a single region. Both populations shared the same primary locus which featured a single haplotype spanning *MTAP* and part of *CDKN2A* which was present on at least one chromosome in 96% of affected dogs, with 65% of cases being homozygous [78]. This haplotype is within a region homologous to human chromosome 9p21 which is an important tumour suppressor locus and is implicated in many human cancers [79, 80]. It is likely that the *MTAP-CDKN2* locus is associated with more than one tumour type; Bernese mountain dogs are also susceptible to lymphoma, mast cell tumours, and osteosarcoma which seem to show familial clustering. However, the presence of the risk haplotype among control dogs could be due to the fact that the risk associated with this locus is modest and that at least some of the control dogs lack additional risk alleles at other loci. After all, it is likely that more than a single locus will be involved in the predisposition [64, 78].

To date there are no published data on whether this haplotype or tumour suppressor locus is important in histiocytic sarcoma in other dog breeds especially rottweilers or golden retrievers. However, there is some evidence that it may be important in other canine sarcomas. Disruption of chromosome 11 involving the loss of the *CDKN2b-CDKN2a* tumour suppressor gene cluster region has been reported in two fibrosarcomas in Labrador retrievers [81]. The same study sequenced exon 1 of *CDKN2B* using DNA from blood of 141 dogs of 18 different breeds and showed widespread polymorphism of this first exon. Seven alleles were recorded and sixteen of the eighteen breeds showed heterozygosity. Further investigations into the role of this tumour suppressor region in other canine soft tissue sarcomas may yield some interesting findings that may start to explain the relatively high incidence of soft tissue sarcomas in dogs in comparison to humans, where such tumours are rare.

## 3. Osteosarcoma 

Osteosarcoma of the long bones is the most common malignant tumour of bone in dogs accounting for 85 −90% of primary bone tumours and almost exclusively affects the large and giant breeds such as rottweiler, great Dane, Irish wolfhound, greyhound, Saint Bernard [82]. The aetiology of osteosarcoma is probably multifactorial; the predilection for the tumour to develop at metaphyseal region of long bones, especially the distal radius and proximal humerus, correlates with weight bearing, and rapid bone growth during early development along with bone stress due to weight bearing (possibly resulting in microfractures) has been implicated in the aetiology of these tumours [83]. Increasing weight and height appear to be important predictive factors for the disease in the dog [84]. Growth hormone has been shown to be present in canine osteosarcoma samples [85] and studies evaluating the role of insulin-like growth factor-1 (IGF-1) and its receptor IGF-1R and hepatocyte growth factor (HGF) and its receptor c-Met in osteosarcoma cell lines and tissues have shown that these factors may contribute to the malignant phenotype [86, 87]. Sex hormones may also contribute to osteosarcoma risk with intact males and females being reported to be at increased risk [84]. However in the rottweiler neutering before 1 year of age appeared to increase risk of bone sarcoma in both male and female dogs [88]. As is the case in many human and canine cancers, alterations in the function of the tumour suppressor genes *RB* and *TP53* have been implicated in the pathogenesis of canine osteosarcoma [89, 90]. The fact that specific breeds appear predisposed suggests that more specific genetic factors may be implicated in the aetiology of canine osteosarcoma. Breeds reported to be at increased risk of developing osteosarcoma include the doberman, German shepherd, golden retriever, great Dane, Irish setter, rottweiler and Saint Bernard [88, 91, 92], large sight-hounds such as Irish wolfhound, Scottish deerhound and Borzoi [84], greyhound, rottweiler and great Dane [83] and Irish wolfhound, Saint Bernard, and Leonberger [93]. It may be argued that these predispositions may be related to size rather than breed, and this is supported by the fact that greyhounds and whippets consistently clustered together in an analysis of molecular variance in microsatellite loci [26, 43], yet whilst appendicular osteosarcoma affects greyhounds [82], the disease is rare in whippets, or indeed in dogs under 25 kg body weight [84, 94]. Interestingly *IGF1* and its associated regions are a major contributing locus in size diversity in dogs, accounting for about 50% of the genetic variation in size [95]. Osteosarcoma is particularly prevalent in retired racing Greyhounds, possibly implicating stress or trauma in the aetiology of the disease in this breed [83, 96]. A familial incidence has been observed in Saint Bernards [91] and more recently a study modelling the transmission of osteosarcoma in a population of over 1000 Scottish deerhounds estimated heritability at 0.69 and modelling suggested that a major gene with dominant effect would explain the pattern of transmission [42].

It is well documented that canine osteosarcoma is a good clinical model for the human disease [97–99] and there is a growing body of evidence to show that canine and human osteosarcoma have a similar molecular pathogenesis [100, 101]. Gene expression profiling of canine osteosarcoma has revealed genes associated with progression, survival, and metastasis that are relevant to human osteosarcoma [100, 102–105]. As is the case in human osteosarcoma, the canine disease is characterised by an extremely complex karyotype indicating extensive genomic instability. Using two breeds of dog with different relative risk of osteosarcoma—rottweiler (12.5% incidence rate) and golden retriever (5% incident rate)—a recent study has shown that the individual genetic background, as defined by breed, influences the tumour karyotype in osteosarcoma [106]. Eleven loci (from 8 different chromosomes) showed a significant difference in the distribution of DNA copy number imbalances between tumours from golden retrievers compared with those from rottweilers; the most significant of these was the deletion of the *WT1* gene which occurred in 48% of the rottweiler cases (14/29) but which was not observed in any of the 9 golden retrievers. Genomic loss of *TP53* and *CDKN2A* suppressor genes were also restricted to rottweilers (7/29 (24%) and 5.29 (17%), resp.). Overall 15/29 rottweilers in this study showed genomic deletion of at least one of the *WT1*, *TP53*, *CDKN2A*, *PTEN,* or *RB1* tumour suppressor genes. These breed-associated imbalances may contribute to or result from heritable risk factors. A larger study which profiled 123 cases of canine osteosarcoma by 1 Mb aCGH also demonstrated a high occurrence of genetic imbalances similar to human osteosarcoma and identified several new candidate genes in regions of the canine genome that had highly recurrent copy number abnormalities [101]. Although this dog population was represented by 4 main breeds, rottweiler (*n* = 34), greyhound (*n* = 25), great Pyrenees (*n* = 13), and golden retriever (*n* = 22), no significant differences were found between aCGH defined regional abberations and breed groups. It is likely that larger cohorts with less variation in tumour histology will be needed to provide the power to detect significance.

## 4. Hemangiosarcoma

Hemangiosarcoma is a highly malignant tumour arising from blood vessels, probably less common than some of the other mesenchymal malignancies, for example, fibrosarcoma; it has been estimated to represent 7% of canine malignant tumours [5] and accounted for 24/100,000 dogs/year in one study [12], which is still considerably higher than angiosarcoma in humans [107]. The most common primary sites for hemangiosarcoma in dogs are visceral organs, notably the spleen and liver; it may also arise in the right atrial appendage. A dermal form of haemangiosarcoma is also seen in dogs, which has a predilection for light haired or nonpigmented skin, particularly on the ventral abdomen and UV light has been implicated in the aetiology of this form of the disease [108]. Visceral hemangiosarcoma has a predilection for certain breeds; the German shepherd dog has been reported to have an increased risk with an odds ratio of 4.7 (95% CI 2.7–7.8) compared to other purebred dogs [109], but boxers and golden retrievers have also been identified as being at increased risk [110, 111]. More recently hemangiosarcoma appears to have become a significant problem in golden retrievers in North America with an estimated life-time risk of 1 in 5 reported by the Golden Retriever Club of America [112, 113]. 

A small number of studies have examined the molecular genetic aspects of canine hemangiosarcoma with respect to growth regulation genes; mutations in the tumour suppressor gene *TP53* have been reported [114, 115] and a further study suggested that alteration of the p16-cyclin D1-Rb pathway may be associated with the pathogenesis of canine hemangiosarcoma. In this study only 18% of samples showed alterations in both *TP53* and p16 [116]. The Rb (p16) pathway is also commonly deregulated in human cancers [117]. As a naturally occurring tumour of endothelial cells, the role of vascular endothelial growth factor (VEGF) in the pathogenesis and progression of hemangiosarcoma has been examined. VEGF has potent angiogenic, mitogenic, and vascular permeability enhancing properties and plays a major role in tumour growth in human cancers where plasma concentrations of VEGF have been shown to correlate with tumour burden and prognosis [118]. In one study, dogs with hemangiosarcoma (*n* = 17) were significantly more likely to have detectable concentrations of plasma VEGF compared to healthy dogs (*n* = 17) [119], although the same group were not able to demonstrate a marked difference in VEGF concentration between body cavity effusions associated with malignant versus nonmalignant diseases [120]. VEGF is a target for some of the newly licensed tyrosine kinase inhibitors including masitinib mesylate (Masivet—AB Science) and it has recently been shown that masitinib causes a dose-dependent-cell death in canine haemangiosarcoma cell lines [121]—further implicating a role for VEGF in canine haemangiosarcoma.

Phosphatase and Tensin Homolog Deleted from Chromosome 10 (*PTEN*) is a tumour suppressor gene which is inactivated in many human cancers [122]. *PTEN* also inhibits angiogenesis, possibly by regulating VEGF gene expression via the P13K pathway [123, 124]. To determine the role of *PTEN* in the origin or progression of canine hemangiosarcoma, Dickerson et al. [125] firstly examined by immunohistochemistry the expression of CD31 (endothelial marker), PTEN, VEGF, p27 AKt, and p-AKT in sections from 12 haemangiosarcomas and 5 benign splenic haematomas and observed some abnormalities in PTEN expression in the tumours. Using cell lines established from some of these tumours the same group identified mutations of *PTEN* in the C-terminal domain that may affect the subcellular localisation and stability of the protein [125]. They speculated that constitutive activation of *P13 K* or loss of *PTEN* function might establish autocrine growth loops that promote autonomous growth and transformation of endothelial cells [122, 126]. However the *PTEN* mutation alone does not fully explain the increased levels of VEGF and other angiogenesis promoting growth factors (platelet derived growth factor and basic fibroblast growth factor) thought to be elaborated by haemangiosarcoma cells, or the role of the inflammatory cells frequently associated with these tumours [125]. Gene expression profiling has shown canine hemangiosarcoma to have a gene signature suggesting that inflammatory and angiogenic pathways play a significant role in its pathogenesis [127]. Genes expressed at significantly higher levels in haemangiosarcomas than osteosarcoma, non-Hodgkin's lymphoma, and leukaemia included *VEGFA*, *TIMP-1*, *FN-1*, *ADAM9*, *PDGFC*, *MMP14*, *TNF*α**, and acid ceramidase.

Gene expression profiling of canine hemangiosarcoma has also shown significant breed differences segregating hemangiosarcomas derived from golden retrievers from hemangiosarcoma in other breeds, with contributions from transcription factors, survival factors, and from pro-inflammatory and angiogenic genes. VEGF Receptor 1 was preferentially enriched in tumours from golden retrievers versus other breeds [113], suggesting that heritable factors mould gene expression phenotypes and consequently biological behavior. The high incidence of hemangiosarcoma in golden retrievers in the USA appears to be a relatively recent phenomenon, as the golden retriever was not reported to be over-represented in a study from the University of Pennsylvania in 1988 [109]. Furthermore hemangiosarcoma does not appear to be particularly prevalent in the breed in the UK, indeed the data from the insured dog study [12] shows golden retrievers to be less at risk of hemangiosarcoma than all other breeds pooled (unpublished data). This is particularly interesting because in a study investigating genetic diversity among four common breeds sampled in the US and Europe, the golden retriever showed a high level of genetic difference between European and American subpopulations allowing them to separate into two distinct populations in clustering analyses corresponding to their geographical origins [128]. Whereas less differentiation was seen in the Bernese mountain dog and very little in rottweilers or flat-coated retrievers from the two continents. This result is partly due to breed popularity and population size. The golden retriever is a very popular breed with more than 42,000 American Kennel Club registrations and 8,000 UK Kennel Club registrations, such that there is a large gene pool on both continents and mixing between the two populations is rare. In contrast the flat-coated retriever popularity in the UK declined following the first world war leaving a small gene pool from which modern dogs derive; the breed still has a relatively small population size worldwide which limits genetic variation within the breed regardless of geographical origin. Not only does this geographic variation in the genetics of certain breeds indicate the importance of taking account of population substructure in genetic studies using worldwide sampling of pure-bred dogs, the genetic differences between the European and North American golden retriever could also be key to understanding the aetiology of hemangiosarcoma in the breed.

## 5. Mast Cell Tumour

Mast cell tumours (MCTs) are common tumours of the canine skin, estimated to represent 7–21 percent of all skin tumours in this species [15, 129, 130]. In the UK, MCT is the second most common canine malignancy, after soft tissue sarcoma with an incidence of 129 per 100,000 insured dogs per year [12]. Cutaneous MCTs are typically solitary lesions but their clinical appearance can be variable and dogs can develop more than one unrelated MCT [131].

The boxer and bull dog breeds including bullmastiffs, Boston terriers, and Staffordshire bull terriers are reported to show an increased risk of developing MCT and it has been postulated that this might be linked to a common ancestry [132]. These breeds have now been shown to cluster closely phylogenetically [26]. Rhodesian ridgebacks, pugs, weimaraners, Labrador retrievers, beagles, and golden retrievers have also been reported to be at increased risk [13]. A phenomenon of occurrence of mast cell tumours in young Shar-Pei dogs was reported from one US veterinary pathology laboratory in 1995. From a total number of 802 submissions diagnosed as MCT, 18 were from Shar-Peis and 5 of these were in dogs less than 2 years of age. Poorly differentiated (Grade III) tumours were more common in younger dogs [133]. Another recently published North American study considered the breed distribution of various canine cutaneous tumours, including MCT, and documented not only those breed over-represented for the disease but also those at reduced risk [130] (Table 3). In our clinic (Cambridge, UK) Labrador retrievers, golden retrievers, and Staffordshire bull terriers all appear over-represented (in comparison to the general Hospital population) whilst German shepherd dogs, cocker spaniels, border collies, cavalier King Charles spaniels, and west Highland white terriers are under-represented [134].

Mast cell tumours show very variable clinical behavior and interestingly this may be influenced by breed. Although boxer dogs and bulldog breeds are at higher risk of developing MCTs, these breeds of dog tend to have low grade, less aggressive tumours, as is also the case for pug dogs [135]. Labrador retrievers tend to have more aggressive tumours and golden retrievers are at risk of developing multiple tumours [131].

Despite a wealth of clinical studies on treatment and prognostic indicators in canine mast cell tumours (review [136]) there is very little published on the molecular genetics of these tumours. Alterations in the p53 tumour suppressor pathway have been identified in some canine MCTs but *TP53* sequencing in a small number of cases did not reveal any mutations [137].

Recent work has implicated the stem cell factor receptor (KIT) as having a role in the aetiology of canine MCTs [138]. KIT is normally expressed on haematopoteiic cells and mast cells. It consists of an extracellular ligand-binding domain, a transmembrane region, and a cytoplasmic tail with tyrosine kinase activity. Activation of the KIT signal transduction pathway plays a role in the growth and development of normal mast cells. KIT is encoded by the protooncogene *c-KIT*, dysregulation of which occurs in many human cancers. In 1996, London and others demonstrated expression of KIT on malignant mast cells derived from 4 spontaneous canine MCTs and subsequently reported internal tandem duplications in exon 11 of *c-KIT* in approximately 30 percent of canine MCTs [139]. Other studies have shown mutations in the juxtamembrane domain of *c-KIT* in dogs with MCTs, mainly within exon 11, with duplications, deletions and substitutions being described [140–146]. Several studies reported a significant association between mutation and higher grade of tumour [142, 143, 145]. To date no mutations have been identified within the canine *c-KIT *gene away from the juxtamembrane domain, and in particular no mutations have ever been identified in the *c-KIT* kinase domain in canine MCT [146]. This is in contrast to mast cell disease in people, where the most common mutation is a single nucleotide substitution resulting in a single amino acid substitution in the kinase domain. Internal tandem duplications have not been reported in the *c-KIT* gene of people.

The presence of *c-KIT *mutations in only a proportion of mast cell tumours suggests that although mutations in this gene may be responsible for the development of some mast cell tumours, it is likely that mutational events in other genes are involved in the carcinogenesis of many mast cell tumours which are yet to be identified. Tumour suppressor in lung cancer-1 (*TSLC1*) is a tumour-suppressor gene coding for an adhesion molecule that is involved in normal mast cell to mast cell, and mast cell to fibroblast interactions [147] Loss of *TSLC1* expression is associated with a poor prognosis in various human tumours, including non-small cell lung cancer, breast, prostate, oesophageal and gastric tumours [148–150] and has been shown to correlate with grade in canine MCTs [151]. In humans, mutations in the mismatch repair (MMR) genes give rise to Lynch syndrome, an inherited predisposition to early onset cancer, especially intestinal adenocarcinoma and skin cancer [152]. MMR expression was investigated immunohistochemically in mast cell tumours from young dogs of predisposed breeds versus old dogs of “non-MCT” predisposed breeds, but no significant differences were observed, suggesting that *MMR* gene defects are not involved in the pathogenesis of canine mast cell tumours [153].

## 6. Lymphoma/Leukaemia

Lymphoma is the most common haematopoietic malignancy in the dog. Dog breed has been shown to play a role in the epidemiology of lymphoma with several studies showing a significantly higher relative risk for boxers, bullmastiff and bulldog breeds compared to other breeds [9, 14, 154, 155]. Other breeds identified with increased relative risk include basset hound, St Bernard, Scottish terrier, Airedale terrier, Bouvier des Flandres, Labrador retriever, and rottweiler [14, 156]. Familial associations with lymphoma have also been reported; nine of 59 bullmastiffs from 3 households died due to lymphoma over a 3-year period with most of the dogs that died also having a close ancestor that had also developed lymphoma [154]. Clustering of lymphoma has also been reported in related rottweilers and three directly related Otterhounds [157].

Interrogation of the data on a population of insured dogs in the UK showed a significant breed effect with the boxer, bulldog, and bull mastiff breeds all having a high incidence of lymphoma in certain age ranges, with the English springer spaniel, golden retriever, and rottweiler breeds showing some indication of excess lymphoma incidence over expectation at certain age ranges—Table 4 [158]. In a study of just over 600 dogs diagnosed with lymphoma in France over 1 year, boxers, setters, and cocker spaniels were significantly over-represented, and a possible predisposition in rottweilers and Beauce shepherd dogs was also reported [159]. These authors also examined distribution of immunophenotype of lymphoma by breed and demonstrated that boxer dogs showed a significant predilection for T-cell lymphoma but were underrepresented among centroblastic polymorphic lymphomas. Other breeds also appeared predisposed to specific immunophenotypes, with B-cell lymphoma predominant in German shepherd dogs and probably in the rottweiler. Whilst this study also demonstrated an association of canine lymphoma with waste incinerators, polluted sites, and radioactive waste, the strong breed associations with immunophenotype and histological subtypes also support a genetic aetiology.

It has recently been shown that the prevalence of subtypes of lymphoma also varies with breed or breed group. A distinct B-cell and T-cell prevalence of lymphoproliferative disease by breed was reported in North America; 1263 dogs representing 87 breeds, whose samples had been submitted for PARR analysis—clonal rearrangement of immunoglobulin heavy chain or T-cell receptor y chain—showed breed-specific susceptibility to develop B-cell or T-cell tumours. Boxers showed increased risk of developing T-cell tumours as did the “Spitz” breeds and Asian “lap” dogs, whereas border collies, basset hounds, cocker spaniels, and dobermans were affected by predominantly B-cell tumours [160]. Because it is retained in related breed groups (e.g., Spitz-type dogs and Asian “lap” dogs) the elevated risk for T-cell lymphoproliferative disease may have arisen ancestrally, whereas increased risk of B cell disease may stem from different risk factors or combinations which arose during the process of breed selection. The boxer T-cell lymphoma has been further classified as being predominantly of TCR-alpha/beta+, CD4+ (helper) T-cells with lymphoblastic (high grade) morphology [161]. This strong breed association with different tumour subtypes may go some way to explaining differences in response to chemotherapy and survival time by breed which have been observed in some clinical studies [162].

Using CGH analysis on a subset of these tumours, the same study [160] identified unique patterns of chromosomal gains and losses that segregated specifically with B-cell tumours and T-cell tumours (as previously identified by [163]) indicating that consistent genetic abnormalities are associated with different tumour types and thus lending weight to the hypothesis that there is a heritable risk for development of canine lymphoma. A deletion of chromosome 14 was exclusively seen in diffuse B-cell lymphoma and occurred in 7 out of 7 golden retrievers but only in 13% (4 of 31) of dogs from other breeds. Thomas et al. [164] have recently reported the results of a genome-wide survey of tumour-associated CNAs through array-based comparative genomic hybridisation analysis in 150 cases of canine non-Hodgkin lymphoma in predominantly three breeds of dog: boxers, Labrador retrievers, and golden retrievers. Interrogation of the recurrent CNAs identified revealed an extensive catalog of chromosomal regions and genes presenting with recurrent DNA copy number imbalance, within which are key genes previously associated with a range of human malignancies; copy number loss of *CDNK2A/B* occurred in 20/36 T-cell lymphoma cases and was more frequent in high grade than low grade cases. The CNA associated most significantly with B cell lymphoma was a highly recurrent deletion of a discrete region on chromosome 26 (74/106 cases) which encompasses the canine immunoglobulin lambda locus. With regard to breed, no significant correlations were found within canine B cell lymphoma suggesting that B cell lymphoma in different breeds shows a highly conserved genomic copy number status, whereas the cytogenetic profiles of T-cell lymphoma were more strongly influenced by the genetic background of the patient. Seven individual loci (on chromosomes 6, 12, 20, and 31) showed highly significant association with breed, each demonstrating an elevated incidence of copy number gain in boxers with T-cell lymphoma [164].

## 7. Melanoma

Melanocytic tumours are relatively common in dogs; they account for 4% of cutaneous tumours [13] and represent one of the most common oral malignancies in the dog [165–167]. Ocular and subungual variants are also described [168]. Canine melanocytic neoplasms vary widely in behavior; oral/mucosal forms are usually malignant and provide a good model for the human mucosal melanoma [4], although a small proportion of tumours at this site are well differentiated and follow a more benign course [169]. Cutaneous and ocular tumours are usually benign, but tumours of the eyelid and nail bed (subungual) are usually malignant. Despite these generalisms, it should be acknowledged that the biological behavior of canine melanocytic neoplasms can vary widely and although many studies have evaluated various prognostic markers, an accurate prognostic classification for these tumours has yet to be established [170, 171]. Cutaneous melanoma occurs more commonly in dogs with heavily pigmented skin, with Schnauzers (both miniature and standard) and Scottish terriers at increased risk [13]. Small breeds especially cocker spaniels and poodles and dogs with heavily pigmented oral mucosa are reported to be at greater risk of oral melanoma [165, 172]. A more recent study of canine oral melanomas showed the Chow Chow, golden retriever, and Pekingese/Poodle mix breeds to be overrepresented, whereas the boxer and German shepherd breeds were under represented [173]. It is not clear whether these breed predilections reflect an underlying genetic risk or merely reflect heavy pigmentation in some breeds, or a combination of the two. However, breed and familial clustering does support underlying genetic risk factors for melanoma [168]. Furthermore, one study has reported that breed may have some prognostic significance with more than 75% of melanocytic neoplasms exhibiting benign behavior in the doberman and miniature Schnauzer, in contrast to more than 85% of melanocytic neoplasms being malignant in the miniature poodle [174], although tumour site may be a confounding factor in these results.

In humans sunlight exposure is an established environmental factor involved in the pathogenesis of cutaneous malignant melanoma [175]; however 6 to 14% of melanoma patients have a family history of melanoma, and it appears there are complex environmental-genetic interactions in such cases. Familial cases tend to be younger, to have a higher number of moles and to develop multiple primary tumours [176]. It has been shown that there are at least two genes involved in familial melanoma in humans, the tumour suppressors *CDKN2A(p16) *and *CDK4*. Families with germline mutation in *CDKN2A* are also prone to pancreatic cancer [177, 178]. A number of pathways and genetic mutations have been identified in nonfamilial cutaneous melanoma; these include activating *BRAF* or *NRAS* mutations resulting in hyperactivation of the mitogen-activated protein kinase (MAPK) pathway [179] and loss or mutation of *PTEN*, a negative regulator of the P13 K pathway [180]. Recent genome wide association studies have identified a number of loci associated with nonfamilial melanoma and many mutations have also been identified through genome sequencing [181–183], but the functional role of these mutations and variants within these loci is not known and it is likely that many other genes and environmental interactions, involved in the pathogenesis of melanoma.

The role of tumour suppressors has been evaluated in canine melanoma. In one study using canine melanoma cell lines and tumour tissue samples, the loss or significant reduction in *p16 *expression was the most common abnormality found in 6/7 cell lines and 21/26 tumour samples [184]. Loss or significant reduction of PTEN expression was also seen in 4/7 cell lines and in 13/27 tumour samples. Changes in other tumour suppressors *TP53*, *Rb,* and *p21* were also detected, suggesting that loss of tumour suppressor function is a common occurrence in canine melanoma. This study included both dermal and oral melanomas; 14 tumours were benign and 11 malignant but the abnormalities in *p16* occurred with equal frequency in both benign and malignant tumours, suggesting that inactivation of this pathway is a critical step in the pathogenesis of melanoma. Loss of the tumour suppressor gene products, p21/Waf1 and p53, has previously been demonstrated in a benign multicentric melanoma from a male Gordon setter [185]. More recently dysregulation of the Wnt/beta catenin signal pathway has been reported in 18 canine cutaneous melanomas, demonstrated by abnormal intracellular accumulation and increased expression of beta catenin [186]. MicroRNA profiles have been examined in canine melanoma tissues and human and canine melanoma cell lines and microRNAs 145 and 205 have been identified as tumour suppressors in both canine and human melanoma cell lines [187, 188]. Other studies have examined KIT expression in cutaneous melanocytic tumours [189] and oral malignant melanomas [190], Cyclooxygenase-2 expression [191, 192], expression of matrix metalloproteinases [193], and Ki67 expression [194] in various canine melanomas but more as prognostic indicators than as clues to the pathogenesis of these tumours. It is clear that canine melanoma offers a relevant model for the human disease and work is ongoing to elucidate further genetic abnormalities that contribute to the pathogenesis of the disease.

## 8. Mammary Tumours

Tumours of the mammary glands are the most common tumour to affect entire bitches representing between 50−70% of all tumour types [18, 195]. A standardised incidence rate of 205/100,000 dogs/year has been reported in a population of insured dogs in the UK [12] and in a Swedish study of insured dogs that the overall rate of mammary tumour development was 111 dogs per 10,000 dog years at risk (DAYR) [196]. Mean age of onset is approximately 8 years. It is well established that ovarian hormone stimulation increases the risk of mammary tumour development in dogs as in other species (including humans) and in the bitch, ovariohysterectomy prior to 2 years of age greatly reduces the risk of mammary tumours in later life [10, 197]. 

The incidence of canine mammary tumours does vary by breed but breeds reported to be at risk vary between different studies and different geographical locations. Poodles (toy and miniature), spaniels (English springer, cocker, and Brittany), Puli, English setter, pointers, German shepherd, Maltese terrier, Yorkshire terrier, and dachshund have all been reported to be predisposed [198]. In the Swedish study where the overall mammary tumour rate was 111 dogs per 10,000 DAYR the English springer spaniel, doberman pinscher, and boxer showed significantly increased incidence rates of 319, 297, and 256 per 10,000 DAYR, respectively, whereas the rough haired collie showed significantly reduced risk of 5 per 10,000 DAYR [196]. It should be noted that very few bitches are routinely neutered in Sweden, so mammary tumours are common [3]. A population based study of mammary tumours in Norwegian dogs showed boxers, cocker spaniels, English springer spaniels, and dachshunds to have the highest relative risk of mammary tumour [195]. A study in Japan reported a lower incidence of malignancy in mammary tumours from small breed dogs [199]. This variation in incidence of mammary tumour risk between breeds suggests a significant heritable genetic component to the disease in dogs. A proportion of human breast cancer is familial; women who have inherited mutations in the *BRCA1* or *BRCA2* (*BRCA 1/2*) genes have substantially increased risk of breast cancer [45, 46] but it is recognised that mutations in these genes only account for a small part, approximately 10%, of the total inherited effect [200]. Furthermore *BRCA1/2* mutation is rare in cases of sporadic breast cancer [201]. Four further genes, *FGFR2, LSP1, MAP3K1,* and *TOX3,* were associated with a mild increase in risk of breast cancer in a GWAS [202]; however over 50% of breast cancers occur in women who do not carry these higher risk genotypes. Breast cancer risk is currently believed to be polygenic with liability conferred by a large number of loci, each contributing a small effect [203, 204]. Oncogenes reported to play an early role in sporadic breast cancer include *MYC*, *CCND1 (Cyclin D),* and *ERBB2 (HER2/neu)* [201]. To date the increasingly powerful molecular techniques available to sequence breast cancer have not been able to elucidate specific genetic solutions but rather have highlighted the substantial genetic diversity underlying this common disease [205].

In contrast to the vast number of gene expression studies in human breast cancer relatively few gene expression studies have been published on canine mammary tumours, most studies having focused on specific genes or receptors. A variable proportion of canine mammary tumours have been reported to contain mutations in *TP53* [206, 207] and studies have shown an association between the level of COX-2 expression, malignant phenotype, and prognosis [208]. A recent comparative study of gene expression in human breast and canine mammary tumours and normal mammary tissue observed a significant overlap of genes deregulated in the tumour samples, as compared to their normal counterparts [209], and pathway analysis of the gene expression data revealed many cancer related pathways, to be similarly perturbed including the P13/AKT, KRAS, PTEN WNT-beta catenin pathways and the MAPK cascade (Table 5). These findings confirm and support the value of canine mammary cancer as a model for human breast carcinogenesis [7, 209]. Although mammary tumours are one of the targets being studies by a European consortium of canine geneticists and clinicians (LUPA), no publications to date have reported differential gene expression underlying susceptibility to mammary cancer by breed [38]. However *BRCA1* and *BRCA2* have been associated with mammary tumours in English springer spaniels in Sweden. Ten human breast cancer genes were evaluated for association with mammary tumours in 212 mammary tumour cases and 143 controls by genotyping SNPs. *BRCA1* and *BRCA2* were significantly associated with mammary tumours and the association was stronger to *BRCA1* in malignant tumours. A borderline association was seen for *FGFR2* [210]. The same group also investigated the role of the dog leucocyte antigen (DLA) system as a genetic risk factor in the aetiology of canine mammary tumours in English springer spaniels, and by genotyping the polymorphic exon 2 of DLA class II loci, identified a significant association between a rare protective haplotype of MCH class II and the incidence of mammary tumours in this population of 363 Spaniels, 218 cases and 145 healthy controls [211]. Not only do these findings support the concept that MHC class II molecules play a critical role in tumour surveillance but that immune response to cancer may be influenced by genotype. A high interbreed and relatively low intrabreed variation in MHC alleles and haplotypes has been documented in over 80 different breeds of dog, and it has been suggested that this variation could provide an explanation for interbreed variation in immune response to vaccines, viruses, and other infections and possibly cancer [212, 213].

## 9. Other Epithelial Malignancies, Carcinomas

In contrast to the high prevalence of lung and large bowel cancer in the human population, particularly in the Western World, carcinomas of the lung and large bowel are relatively uncommon in the canine population. A number of tumour registries and clinical case studies have highlighted breed predispositions for carcinomas arising at other sites, as listed in Table 6. Many of these are really just anecdotal observations, the underlying genetic basis of which has rarely been investigated; however a few are worthy of note.

Canine anal sac gland carcinoma (ASGC) is a relatively uncommon malignancy arising from the apocrine glands in the walls of the anal sacs. This tumour is invasive and metastatic in nature and is often associated with a paraneoplastic hypercalcaemia. Although ASGC may arise in any breed of dog the English cocker spaniel and to a lesser degree other spaniel types (English springer and cavalier King Charles) have been reported to be predisposed to development of this tumour [214]. Predisposing genetic factors have yet to be elucidated but an association between ASGC and dog leucocyte antigen DQB1 has been demonstrated in the English cocker spaniel [215]. The allele distribution in DLA loci DAL-DRB1, -DQA1, and DQB1 was compared between 42 cases and 75 controls; there was no difference in allele distribution in DLA-DRB1 while a significant difference was obtained for DLA-DQA1 and DQB1 alleles, with the DLA-DQB1-00701 allele having a higher frequency in cases than controls [215]. Interestingly a similar DLA-DQB1 allele association has been shown in the English cocker spaniel and immune-mediated hemolytic anaemia [216]. It is not known at this time whether the allele itself has a causative effect on development of disease, perhaps through altered immune function, or whether it is an indirect association as a marker locus with a causative locus located in its vicinity.

Other breeds or types of dog have been associated with less common carcinomas, for example, squamous cell carcinoma of the digit appears to have a predilection for large black dogs including giant Schnauzers and standard poodles [217, 218], who interestingly cluster quite closely on the phylogenetic tree [26]. A familial association has been reported in giant schnauzers [219]. Scottish Terriers have been shown to be at 19-fold increased risk of transitional cell carcinoma of the bladder compared with mixed breeds, the cause of which is not known but perhaps represents genetic predisposition through differences in metabolic and detoxification pathways [220]. In addition to the Scottish terrier, Airedale terriers and beagles have also been identified as being predisposed to lower urinary tract tumours, whereas German shepherd dogs appear to be under-represented [221]. In contrast to human prostatic carcinoma, which is androgen dependent, prostate cancer has been reported to occur more commonly in neutered than intact male dogs, possibly because a high proportion of canine prostatic carcinomas are transitional cell origin as opposed to adenocarcinoma [222]. Although neutering status is a strong risk factor for canine prostatic cancer, breed predisposition has been demonstrated with dobermann pinschers, Shetland sheepdogs, Scottish terriers, beagles, German shorthaired pointers, Airedale terriers, and Norwegian elkhounds all having a statistically significantly increased odds of having prostatic cancer of any histology independent of neutering status [11].

## 10. Brain Tumours

Intracranial neoplasia is quite well described in the dog where the most common primary CNS tumour is meningioma, followed by glial tumours (astrocytoma and oligodendroglioma). Choroids plexus tumours, medulloblastoma, neuroblastoma, and ependymomas occur less frequently. In a postmortem study of 173 dogs, golden retrievers and boxers were at increased risk to develop primary intracranial tumours relative to their frequency in the general Hospital population [223]. Brachycephalic breeds have previously been reported to be prone to development of glioma, but in this study only boxers and Boston terriers were more likely to have an astrocytoma, oligodenroglioma, or undifferentiated glioma than another type of primary intracranial neoplasm. Boxers account for nearly 50% of dogs with brain tumours presented for radiotherapy at the Cancer Therapy Unit, Cambridge, and appear equally affected by glioma and pituitary macroadenoma (unpublished data).

## 11. Multiple Primary Tumours

This paper has highlighted strong breed predispositions to certain types of cancer and breeds that are prone to more than one tumour type. In reviewing the more recent gene expression literature it is apparent that tumour suppressor genes, particularly *CDKN2A*, *CDNK2B,* and *PTEN,* are implicated in the pathogenesis of many canine tumours, but to date there is little evidence to demonstrate that defects in these pathways are inherited in the susceptible breeds. Recently a germline mutation in the mesenchymal-epithelial transition factor (*MET*) protooncogene was found in approximately 70% of rottweiler dogs, a breed predisposed to several types of cancer [224]. This supports the concept that particular dog breeds may carry germline mutations that contribute to high rates of cancer in a manner similar to heritable, cancer-associated mutations in humans. Inherited defects in tumour suppressor genes have been associated with increased risk of early onset cancer and development of multiple primary tumours in humans, notably *TP53* and Li-Fraumeni syndrome [44]. The literature contains a number of case reports of multiple tumours in individual dogs, for example, simultaneous aortic body tumour and pulmonary histiocytic sarcoma in a flat-coated retriever [225], but only recently has a detailed analysis of dogs presenting with multiple distinct types of neoplasia been published [226]. These represented just 3%, 53 of 1722 dogs presented to the oncology service at Colorado State University Veterinary Medical Centre; although no breed or sex predisposition was apparent, dogs with mast cell tumour, malignant melanoma, and thyroid carcinoma were significantly over-represented.

## 12. Conclusions

Most of this review has focused discussion on the breeds of dog associated with an increased risk of developing cancer and breed associations recognised in common tumours. It should be acknowledged that for most forms of cancer the aetiology is likely to be multifactorial and although genetics are important environmental factors such as chemical exposure [227] and hormonal/metabolic factors have been shown to increase the risk of development of certain tumours. 

The domestic dog has been bred selectively for many years to accentuate traits that are desirable in the eyes of the breeder. Each dog breed with a specific pool of alleles represents a genetic isolate, facilitating the identification of susceptibility alleles in dogs breeds as compared to humans. Pure-bred dogs allow the identification of rare variants in the whole canine population because they have been accidentally selected in a given dog breed, and for multifactorial diseases such as cancer, the impact of environmental exposure can be analysed against a reasonably homogenous genetic background. Thus the fact that different breeds of dog have different predilections to different forms of cancer is not only interesting but could provide a very important insight into the genetic aetiology of many forms of the disease. This is particularly important in a disease like cancer, where the complex disease phenotypes are likely to have developed from a combination of multiple genetic risk factors, each with relatively weak penetrance. It could be that the genetic architecture of cancer in predisposed breeds such as the Bernese, boxer, and golden retriever is basically the same as in other breeds with other tumours. The difference is that in the former, the risk conferred by one or more predisposing alleles could be higher. Whether the same alleles predispose to various types of cancer in Bernese, boxer, and golden retriever, or whether different loci, segregating in each one of those breeds, are responsible for the different tumour types, is an interesting question which remains to be answered.

 Much has been made of the many problems associated with pedigree dogs in the popular and veterinary press [228, 229]; it is therefore somewhat ironic that as a result of these problems the pedigree dog provides an ideal model to identify phenotype/genotype relationships relevant to human disease.

The sequencing of the canine genome [29] along with the new genomic tools and resources now available for the study of the dog has allowed workers to start to analyse complex diseases such as cancer. As this review has shown, the dog is already proving to be a valuable model for this purpose and further well designed and conducted population based studies into breed-related canine cancers would provide an important platform to take forward future genetic research.

## Figures and Tables

**Table 1:** Proportional cancer-related mortality by breed. Based on data from [1], cancer accounted for 27% of deaths (4282 of 15,881).

Breed	All deaths	Cancer-related death			Median age at death
		*N *	%	95% CI	
Irish water spaniel	95	53	55.8	45.8–65.8	9.33
Flat-coated retriever	610	331	50.3	50.3–58.2	9.83
Hungarian wirehaired vizsla	15	7	46.7	21.4–71.9	9.83
Bernese mountain dog	394	180	45.7	40.8–50.6	8.0
Rottweiler	137	62	45.3	36.9–53.6	8.92
Italian spinone	47	21	44.7	30.5–58.9	9.0
Leonberger	47	21	44.7	30.5–58.9	7.08
Staffordshire bull terrier	117	52	44.4	35.4–53.4	12.75
Welsh terrier	23	10	43.5	23.3–63.7	12.67
Giant schnauzer	39	16	41	25.6–56.6	10.0
Airedale terrier	81	32	39.5	28.9–50.2	10.75
Golden retriever	927	360	38.8	35.7–42.0	12.25
Boxer	130	50	38.5	30.1–46.8	10.25
Briard	71	27	38.0	26.7–49.3	11.17
French bulldog	71	27	38.0	26.7–49.3	9.0
Bullmastiff	96	36	37.5	27.8–47.2	7.46
Alaskan Malamute	14	5	35.7	10.6–60.8	10.71
Saluki/gazelle hound	132	47	35.6	27.4–43.8	12.0
Nova Scotia duck tolling retriever	9	3	33.3	2.5–64.1	8.0
Basset griffon vendeen	76	25	32.9	22.3–43.5	12.04
Beagle	241	79	32.8	26.9–38.7	12.67
English setter	384	126	32.8	28.1–37.5	11.58
Norwegian elkhound	71	23	32.4	21.5–43.3	13.17
Siberian Husky	129	41	31.8	23.7–39.8	12.58
Keeshond	104	33	31.7	22.8–40.7	12.21
Tibetan terrier	95	30	31.6	22.2–40.9	12.17
Basset hound	142	44	31.0	23.4–38.6	11.29
Labrador retriever	574	179	31.2	27.4–35.0	12.25
Afghan hound	143	44	30.8	23.2–38.3	11.92
Rhodesian ridgeback	183	56	30.6	23.9–37.3	11.0
Irish red and white setter	179	54	30.2	23.4–36.9	11.42
Standard poodle	118	35	29.7	21.4–37.9	12.0
German shorthaired pointer	159	47	29.6	22.5–36.7	12.0
Cocker spaniel/English cocker	289	85	29.4	24.2–34.7	11.17
Field spaniel	68	20	29.4	18.6–40.2	11.63
Welsh corgi Pembroke	116	33	28.4	20.2–36.7	12.21
Welsh corgi cardigan	53	15	28.3	16.2–40.4	12.17
Gordon setter	157	46	29.3	22.2–36.4	11.08
Irish setter	451	123	27.3	23.3–31.4	12.0
Newfoundland	269	73	27.1	21.8–32.5	9.67

Welsh springer spaniel	157	42	26.8	19.8–33.7	12.58
English springer spaniel	90	24	26.7	17.5–35.8	12.0
Lancashire heeler	30	8	26.7	10.8–42.5	11.75
Samoyed	223	59	26.5	20.7–32.2	12.5
Doberman	100	26	26	17.4–34.6	10.5
Soft coated wheaten terrier	111	29	26.1	18.0–34.3	12.5
Large Munsterlander	69	17	24.6	14.5–34.8	11.33
German wirehaired pointer	41	10	24.4	11.2–37.5	10.0
Weimaraner	242	58	24.0	18.6–29.3	11.13
Border collie	106	25	23.6	15.5–31.7	12.25
Tibetan spaniel	125	29	23.2	15.8–30.6	14.42
Belgian shepherd	113	26	23.0	15.2–30.8	12.5
Bull terrier	209	48	23.0	17.3–28.7	10.0
Dandy Dinmont terrier	62	14	22.6	12.2–33.0	12.17
Shetland sheepdog	365	81	22.3	18.0–26.5	12.5
Manchester terrier	32	7	21.9	7.6–36.2	12.83
Norwich terrier	56	12	21.4	10.7–32.2	13.38
Miniature schnauzer	214	46	21.5	16.0–27.0	12.08
Pointer	145	30	20.7	14.1–27.3	12.42
Finnish spitz	42	9	21.4	9.0–33.8	11.13
Bearded collie	278	54	19.4	14.8–24.1	13.5
Cairn terrier	124	24	19.4	12.4–26.3	14.0
Dalmatian	199	38	19.1	13.6–24.6	12.67
Border terrier	177	34	19.2	13.4–25.0	14.0
Sussex spaniel	42	8	19.0	7.2–30.9	11.13
Deerhound	287	54	18.8	14.3–23.3	8.6
Bulldog/British bulldog	180	33	18.3	12.7–24.0	6.29
Lhasa Apso	84	15	17.9	9.7–26.0	14.33
Dachshund (all)	245	41	16.7	12.1–21.4	10.75
German spitz/klein or mittel	43	7	16.3	5.2–27.3	11.33
Shih tzu	83	12	14.5	6.9–22.0	13.17
Other breeds (*n* = 93)	4524	806	17.8		

Total	15,881	4282	27.0		

**Table 2:** Adapted from [23] Table 5, percentage of deaths due to cancer suffered by dogs of different breeds compared with the percentage of the breed in the survey population.

Breed	% chance of dying of cancer	% in survey population	Ratio	Tumour types for which breed has been reported to be at risk
Overrepresented				
Irish wolfhound	0.89	0.31	2.9	Osteosarcoma
Rottweiler	7.35	3.53	2.1	Osteosarcoma, histiocytic sarcoma, lymphoma
Afghan hound	0.67	0.38	1.8	Osteosarcoma
Standard poodle	1.34	0.8	1.7	SCC digit
Weimaraner	1.34	0.8	1.7	Mast cell tumour
Irish red and white setter	0.90	0.7	1.3	
Staffordshire bull terrier	1.78	1.4	1.3	Mast cell tumour
Boxer	4.45	3.35	1.3	Mast cell tumour, glioma
Cairn terrier	1.34	1.12	1.2	
Old English sheepdog	2.00	1.61	1.2	
Golden retriever	8.91	7.16	1.2	Mast cell tumour, lymphoma, oral melanoma, fibrosarcoma, histiocytic tumours
Flat-coated retriever	0.67	0.56	1.2	Histiocytic sarcoma
Average				
Dobermann	2.67	2.48	1.1	
English springer spaniel	3.79	3.63	1.0	
Labrador retriever	11.58	11.45	1.0	Mast cell tumour
Great Dane	1.34	1.54	0.9	Osteosarcoma
Underrepresented				
Border collie	1.56	2.02	0.8	
Cocker spaniel	3.12	3.73	0.8	Anal gland adenocarcinoma
Crossbred	13.36	16.58	0.8	
German shepherd	8.46	10.02	0.8	Haemangiosarcoma
West Highland white terrier	2.00	2.79	0.7	
Shetland sheepdog	0.89	1.40	0.6	
Yorkshire terrier	1.34	2.2	0.6	
Jack Russell	1.34	2.62	0.5	
Rough Collie	0.67	1.78	0.4	Gastric carcinoma
Bulldog	0.22	0.59	0.4	Mast cell tumour, glioma
Welsh springer spaniel	0.22	0.52	0.4	
Airedale	0.22	0.63	0.3	
Irish setter	0.22	0.7	0.3	
Dachshund	0.22	1.43	0.2	
Cavalier King Charles spaniel	0.45	2.06	0.2	
Beagle	0	0.56	0	Mammary tumours

**Table 3:** Breeds over- and underrepresented in a recent American survey of breed association to canine mast cell tumours [130].

Dogs overrepresented	Dogs underrepresented
Boxer	German shepherd dog collie
Rhodesian ridgeback	Toy Poodle
Vizsla	Chihuahua
Boston terrier	Lhasa Apso
Weimaraner	Miniature poodle
Chinese Shar-Pei	Siberian Husky
Bullmastiff	Yorkshire terrier
Dutch Pug	Rottweiler
Labrador retriever	Great Dane
American Staffordshire terrier	Doberman pinscher
Golden retriever	Dachshund
English setter	American cocker spaniel
English pointer	Mixed

**Table 4:** Patterns of excess lymphoma by breed and age, comparing the observed numbers of lymphoma cases per breed, over four (quartile) age ranges, with expected numbers computed from age incidence.

	Age ≤ 3		4 ≤ Age ≤ 6		7 ≤ Age ≤ 9		10 ≤ Age ≤ 14		No dogs by breed
	O	E	O	E	O	E	O	E	
Border collie	1	0.37	0	0.47	1	0.68	1	0.93	2378
Boxer	1	1.06	2	1.05	4*	**0.75 **	3^¶^	**0.52 **	5628
Bulldog	3*	**0.38 **	0	0.24	0	0.10	0	0.006	1720
Bullmastiff	0	0.21	3^§^	**0.17 **	0	0.09	0	0.016	1075
CKCS	0	0.71	1	1.15	2	1.08	0	0.85	4529
Cocker spaniel	0	0.95	1	1.16	1	1.06	0	0.90	5568
Crossbred	2	1.01	1	2.06	4	4.03	4	5.13	8855
Dalmatian	1	0.37	0	0.34	0	0.20	0	0.20	1973
Doberman	1	0.34	0	0.30	0	0.43	1	0.54	2006
English springer spaniel	0	0.72	0	0.87	1	0.89	3	0.93	4308
German shepherd dog	0	2.14	1	2.36	1	2.33	1	1.82	12157
Golden retriever	5^¶^	**1.02 **	3	2.78	1	2.40	6	2.86	11348
Irish setter	0	0.16	1^¶^	**0.31 **	0	0.22	0	0.26	1123
Labrador retriever	2	2.84	2	3.20	2	2.89	1	3.02	16259
Miniature schnauzer	0	0.17	0	0.20	1	0.18	0	0.16	1007
Old English sheepdog	0	0.18	1	0.29	1	0.27	0	0.26	1086
Rottweiler	0	0.45	0	0.32	2	0.44	0	0.38	2446
Staffordshire bull terrier	0	0.51	0	0.39	1	0.47	1	0.38	2844
West Highland white terrier	0	1.07	0	1.15	0	0.99	1	0.81	6134
Other breeds	6	6.5	11	8.22	5	7.48	5	7.05	38240

Total									130684

O: observed, E: expected.

*Individually *P* ≤ 0.01 by Poisson distribution.

^§^Individually *P* ≤ 0.001 by Poisson distribution.

^¶^Individually *P* ≤ 0.05 by Poisson distribution.

Table from [158].

**Table 5:** Comparative analysis of the role of critical genes and signalling pathways involved in the carcinogenesis of human breast cancer and canine mammary tumor.

	Human breast cancer	Canine mammary tumor
Gene sets/signaling pathways		
P13K/AKT	Upregulation	Upregulation
KRAS	Upregulation	Upregulation
PTEN	Downregulation	Downregulation
Wnt*b catenin	Upregulation	Upregulation
MAPK cascade	Upregulation	Upregulation
BRCA1	Downregulation	Downregulation
BRCA2	Upregulation	Upregulation
P53	Downregulation	Downregulation

Table modified from Pinho et al., 2012 [7], and based mainly on data from Uva et al., 2009 [209].

**Table 6:** Additional breed predilections to assorted tumor types.

Tumour type	Breeds showing predilection	References
Anal sac gland carcinoma	English cocker spaniel	[214]
	English springer spaniel	
	Cavalier King Charles	

Squamous cell carcinoma digit	Giant schnauzer	[217]
	Standard poodle	[219]

Transitional cell carcinoma, bladder	Scottish terrier	[220]
	West Highland white terrier	
Lower UT carcinoma	Shetland sheepdog	[221]
	Airedale terrier	
	Beagle	

Prostatic carcinoma	Doberman pinscher	[11]
	Shetland sheepdog Scottish terrier	
	Beagle German short haired pointer Airedale terrier	
	Norwegian elkhound	

Gastric carcinoma	Rough Collie	[230]
	Belgian shepherd	[41]

Thyroid carcinoma	Golden retriever	[231]
	Beagle	
	Siberian Husky	

Nasal cavity carcinoma	Collie	[232]
	Shetland sheepdog	

Aortic/Carotid body tumors (paraganglioma)	English bulldog	[233]
	Boxer	
	Boston terrier	

Brain tumors	Boxer	[223]
	Golden retriever	
	Boston terrier	

Testicular tumors		[234]
Sertoli cell tumor	Shetland sheepdog	
	Collie	
Seminoma	Norwegian elkhound	

## Abbreviations

GWAS: Genome wide association study/ies
CNA: Copy number abnormality
TCR: T-cell receptor
PARR: PCR for antigen receptor rearrangements
PCR: Polymerase chain reaction
VEGF: Vascular endothelial growth factor
CGH: Comparative genomic hybridization
SNP: Single nucleotide polymorphism
DLA: Dog leucocyte antigen
MHC: Major histocompatibility complex
ASGC: Anal sac gland carcinoma
GRCA: Golden retriever club of america
DYAR: Dog years at risk.
